# Inferring Adaptive Codon Preference to Understand Sources of Selection Shaping Codon Usage Bias

**DOI:** 10.1093/molbev/msab099

**Published:** 2021-04-19

**Authors:** Janaina Lima de Oliveira, Atahualpa Castillo Morales, Laurence D Hurst, Araxi O Urrutia, Christopher R L Thompson, Jason B Wolf

**Affiliations:** 1 Instituto de Biologia, Universidade Federal da Bahia, Salvador, Bahia, 40170-115, Brazil; 2 Milner Centre for Evolution and Department of Biology and Biochemistry, University of Bath, Claverton Down, Bath, BA2 7AY, UK; 3 Instituto de Ecologia, UNAM, Ciudad de Mexico 04510, Mexico; 4 Centre for Life's Origins and Evolution, Department of Genetics, Evolution and Environment, University College London, Darwin Building, Gower Street, London, WC1E 6BT, UK

**Keywords:** codon usage bias, biased gene conversion, weak selection, translation

## Abstract

Alternative synonymous codons are often used at unequal frequencies. Classically, studies of such codon usage bias (CUB) attempted to separate the impact of neutral from selective forces by assuming that deviations from a predicted neutral equilibrium capture selection. However, GC-biased gene conversion (gBGC) can also cause deviation from a neutral null. Alternatively, selection has been inferred from CUB in highly expressed genes, but the accuracy of this approach has not been extensively tested, and gBGC can interfere with such extrapolations (e.g., if expression and gene conversion rates covary). It is therefore critical to examine deviations from a mutational null in a species with no gBGC. To achieve this goal, we implement such an analysis in the highly AT rich genome of *Dictyostelium discoideum*, where we find no evidence of gBGC. We infer neutral CUB under mutational equilibrium to quantify “adaptive codon preference,” a nontautologous genome wide quantitative measure of the relative selection strength driving CUB. We observe signatures of purifying selection consistent with selection favoring adaptive codon preference. Preferred codons are not GC rich, underscoring the independence from gBGC. Expression-associated “preference” largely matches adaptive codon preference but does not wholly capture the influence of selection shaping patterns across all genes, suggesting selective constraints associated specifically with high expression. We observe patterns consistent with effects on mRNA translation and stability shaping adaptive codon preference. Thus, our approach to quantifying adaptive codon preference provides a framework for inferring the sources of selection that shape CUB across different contexts within the genome.

## Introduction

Codon usage bias (CUB), where synonymous codons are not used in equal frequencies, is well documented across species from all three domains of life (Duret 2002; Chen et al. 2004). Alternative explanations for CUB have played a central role in the decades long neutralist–selectionist debate. On the one hand, a purely neutral framework has been proposed in which CUB arises simply as a consequence of mutational bias (Kimura 1968; King and Jukes 1969; Sueoka 1988; Palidwor et al. 2010). This framework can explain a large proportion of variation in CUB across taxa, including bacteria, archaea, plants, and animals, where mutational bias has presumably shaped GC% at third codon positions (GC3) to vary from near zero to nearly 100% across species (Knight et al. 2001; Palidwor et al. 2010). Across this range, we see a corresponding shift in CUB such that the use of the same codon can go from near zero to near 100% use as genomic composition changes (see Knight et al. 2001). This process can even explain intragenomic (between gene) variation if there are different mutational biases in different genomic regions (Wolfe et al. 1989; Wolfe 1991; Duret and Galtier 2009; Jinks-Robertson and Bhagwat 2014). On the other hand, it has been proposed that selection operates on synonymous mutations because they affect a variety of processes, some linked to transcription and translation (Ikemura 1981; Ikemura 1982; Stoletzki and Eyre-Walker 2007; Plotkin and Kudla 2011; Zhou et al. 2016). For example, the translational selection model is often invoked to explain observations where commonly used codons match the most abundant tRNAs (Ikemura 1981; Ikemura 1982; Bulmer 1991; Sharp et al. 1995; Duret 2002). More generally, transgenes with “foreign” codon usage profiles (i.e., which deviate strongly from the endogenous pattern) often show reduced transcription or translation rates (Kudla et al. 2006; Plotkin and Kudla 2011; Mordstein et al. 2020). Functional support for the selectionist perspective also comes from the discovery of synonymous mutations that cause disease (Chamary et al. 2006; Sauna and Kimchi-Sarfaty 2011; Sauna and Kimchi‐Sarfaty 2013), which also indicates the importance of identifying and understanding the drivers underlying adaptive codon use.

One way to distinguish between neutral and selectionist models is to develop a framework that can accurately identify the action of selection in the presence of neutral/mutational bias. In this framework, deviations from the neutral pattern of CUB would capture the signatures of natural selection driving adaptative CUB (Knight et al. 2001; Palidwor et al. 2010). Therefore, this framework relies on the ability to accurately define an unbiased neutral expectation such that deviations from neutrality can be accurately characterized (Eyre-Walker 1993). One approach is to measure nucleotide content in genomic domains assumed to evolve neutrally, such as in introns, intergenic regions, and pseudogenes or use nucleotide biases at some codon third sites to predict others (Karlin and Mrázek 1996; Urrutia and Hurst 2001). In principle, between species comparisons (e.g., using nucleotide triplets) can allow substitutional processes to be inferred, again often using sequences predefined as neutrally evolving.

An alternative is to make no assumptions about mutation bias but instead to presume that selection must be stronger in highly expressed genes. The codon usage profile of the most highly expressed genes is then used to infer the optimal codon set and thence the degree of codon adaptation of any given gene, measured in terms of the gene’s usage of the “optimal” codon set (Sharp and Li 1987). However, it is possible that genes with lower expression are also under selection to optimize their ability to produce sufficient protein product (e.g., mRNA stability). Importantly, this pattern of selection may deviate from that in the most highly expressed genes, which may themselves experience very specific selective constraints that shape codon usage that are not imposed on most other genes in the genome, as supported by transgene studies (Kudla et al. 2006; Mordstein et al. 2020). Hence, inferring adaptive codon preference from the profile of the most highly expressed genes could potentially reflect a tautology that fails to capture the more general profile of natural selection that shapes codon use in most genes in the genome. Nonetheless, such a methodology has claimed support from observations that synonymous rates of evolution are lower in genes with high CUB (Shields et al. 1988; Sharp and Li 1989; Moriyama and Hartl 1993; Powell and Moriyama 1997). However, this conclusion may well, at least in some incidences, be an artifact of inaccurate estimation methodology that is itself skewed by CUB (Dunn et al. 2001).

Although these methods appear logical, GC-biased gene conversion (gBGC) presents a major challenge (Marais 2003; Duret and Galtier 2009; Galtier et al. 2018). In this process, an AT:GC mismatch is preferentially repaired in favor of the GC residue during heteroduplex mismatch repair through meiotic double strand break repair. For example, in the human genome A:G mismatches are repaired to G about 70% of the time during noncrossover recombination events (giving CG) (Halldorsson et al. 2016). gBGC appears to be an important force that can mimic the influence of selection (Gutz and Leslie 1976), but which can operate on any sequence, regardless of whether it is considered to be neutral or under selection (Glémin et al. 2015). Importantly, the level of recombination also increases with GC content (Eyre-Walker 1993; Fullerton et al. 2001), which may be a consequence of gBGC (Eyre-Walker 1993) or caused by high GC (Marsolier-Kergoat and Yeramian 2009; Kiktev et al. 2018). Regionalization of recombination hot and cold spots will, therefore, result in a correlation between GC3, the GC composition of introns, and the GC content of flanking sequence (Duret and Galtier 2009). There is also evidence for AT → GC fixation bias in GC-rich/high-recombining domains (Duret et al. 2002; Lercher et al. 2002). These factors are likely to have led to the evolution of isochores, which means that the best prediction of CUB in the human genome is not a property of the gene but rather, the isochore within which the gene is found (D’Onofrio et al. 1991; Eyre-Walker and Hurst 2001; Urrutia and Hurst 2001).

The presence of gBGC represents a major problem for studies that rely upon measures of observed nucleotide content or substitutional profiles in putatively neutral sequence to provide a measure of mutational bias, as neither will reflect the underlying mutational process, even if the sequence class is neutrally evolving (when mutations are not subject to gBGC). Similarly, even if highly expressed genes are indeed subject to stronger selection, it is also possible that these genes show a shift in GC content independent of selection because they are more prone to strong gene conversion, perhaps due to being in domains of high recombination. Indeed, in humans, highly expressed genes tend to be GC rich (Kudla et al. 2006; Mordstein et al. 2020) and located in GC-rich isochores (Lercher et al. 2003) with high recombination rates (Fullerton et al. 2001). Given this, basing inference of adaptive codon preference on CUB in the most highly expressed genes could potentially be misleading. Attempts have been made to correct the substitutional profiles to account for biased gene conversion (Galtier et al. 2018), for example, by examining A ↔ T, G ↔ C changes separately from AT ↔ GC changes. With transgene evidence indicating that high GC confers higher expression levels (Kudla et al. 2006; Mordstein et al. 2020; Zuckerman et al. 2020), these methods are problematic in ascribing all AT → GC fixation events to gBGC, potentially overlooking a major source of selection.

An alternative method to determine skew from mutational expectations is one in which the mutational bias is more directly observed. Perhaps the “gold standard” is to have data from parent offspring trios. However, to date this only exists for a handful of species and can be noisy because this method usually only identifies a few mutations per trio, especially in species with small genomes. False-positive mutational inference is hence a problem for such methods. Consequently, mutation accumulation lines (Long et al. 2018) or analyses of mutational profiles based on rare segregating single nucleotide polymorphisms (SNPs; Hershberg and Petrov 2010; Hildebrand et al. 2010; Charneski et al. 2011) are more commonly used. For example, such studies have revealed that the mutational process is GC → AT biased in humans, which results in a predicted neutral mutational equilibrium that is strongly AT biased (Smith et al. 2018). This is also approximately independent of the GC content of the isochore (Smith et al. 2018), which suggests that codon usage is GC biased due to a fixation bias, rather than a mutation bias. More generally, when mutation-derived equilibria are compared against observed GC content, most genomes show higher GC3 than the neutral mutational predicted equilibrium GC (Long et al. 2018).

Even if we can infer the mutational process in a manner that is not confounded by gBGC, it remains very difficult to show that observed compositional profile deviations from a mutational null are due to selection. More generally, we can identify fixation bias as a deviation from mutational null (Lercher et al. 2002). Moreover, even if the fixation bias appears adaptive because of its match to the tRNA pool, it is possible that the persistent influence of biased gene conversion skews codon usage and tRNA pools adapt to this skew not vice versa.

Given the potential impact of various nonadaptive processes that can bias base composition, we suggest that it might be most instructive to examine patterns of CUB in species in which the role of gBGC is a priori thought to be minimal. This would allow for the impact of natural selection on CUB to be more cleanly characterized and the validity of common functional assumptions to be tested in a nontautologous fashion (e.g., whether preference inferred from the most highly expressed genes represents an adaptation that applies across most genes). Here we exploit the social amoeba *Dictyostelium discoideum* to implement such an analysis.

*Dictyostelium discoideum* offers a compelling system for a number of reasons. The impact of gene conversion can be particularly problematic for inferences of selection on codon usage, but, although it is typically profound in mammals (Halldorsson et al. 2016) and birds (Smeds et al. 2016), this is rarely the case in single-celled species (Liu et al. 2018). For example, the net bias in yeast has been estimated from tetrad analysis to be weakly (0.03%) in favor (but not significantly so) of AT → GC repairs (Liu et al. 2018). Second, it has one of the most extreme base composition biases recorded for eukaryotes to date (∼22.4% GC; Eichinger et al. 2005), second only to the human malaria parasite *Paramecium falciparum* (∼19.4% GC; Gardner et al. 2002). It is logical, therefore, to assume that the impact of gBGC to counteract the strong GC → AT-biased mutational profile must be small. Moreover, the extreme base composition bias provides for a clear and strong expectation for the neutral pattern of CUB driven by mutation–drift. Finally, we can exploit the available genome sequences from 67 strains that enable analyses of patterns of SNP variation (de Oliveira et al. 2019), which allow for inference of mutational profiles and rates and the signatures of selection on synonymous changes. We can also exploit the extensive expression data available, which allows us to characterize how patterns of CUB relate to the expression properties of genes.

To achieve this goal, we first investigate whether we can discern any of the classical fingerprints of gBGC conversion in *D. discoideum*, namely a GC-recombination correlation and local GC correlations (GC3 vs. GC intron, GC3 vs. intergenic GC). Having found no coherent evidence of gBGC (or any effects are miniscule), we next defined the mutation profile (at mono and dinucleotide level of resolution) from SNPs, which allows us to infer the expected neutral pattern of codon usage. As expected (Long et al. 2018), AT mutation bias dominates the pattern of CUB. However, accounting for the neutral pattern allows us to infer the influence of selection driving the relative use of synonymous codons away from the neutral expectation, which we interpret as providing a measure of the direction and strength of selection. Using these measures of “adaptive codon preference,” we test whether classical expectations of drivers of CUB hold and whether the pattern differs from what would be inferred using alternative approaches. In particular, we ask whether we can recover the expected correlation between our measure of adaptive codon preference and levels of gene expression, which provides a rare direct test of the hypothesis that selection is stronger in highly expressed genes. To understand how the pattern of selection on codon usage varies by context, we compare the patterns of adaptive codon preference with the shifts in CUB associated with levels and conditionality of gene expression and with variation in codon position within genes (in terms of relative distance from start and stop site). Together our results show that deviations from the neutral pattern of codon use provide a clear inference for how selection shapes the use of alternative codons and how this pattern of selection changes across contexts. Finally, we show that this pattern is similar to but deviates from the pattern that would be inferred solely based on the association with high expression.

## Results

### gBGC Has Little Impact on GC Composition in *D. discoideum*

In mammals, local biased gene conversion hotspots can cause CUB to vary across the genome and reflect the local composition in the genomic region where it is found (D’Onofrio et al. 1991; Urrutia and Hurst 2001). To determine whether gBGC also introduces heterogeneity into patterns of codon usage across the *D. discoideum* genome, we implemented five complementary analyses. In the first, we used a sliding window analysis to examine variation in GC content across the genome. GC content is largely evenly distributed across all chromosomes apart from a few peaks that are associated with genomic regions enriched in transposable elements (TEs; supplementary fig. S1, Supplementary Material online). After removing the areas from small sections of chromosomes 1 (bases 1–200 kb) and 6 (bases 850–900 kb), we find that there is no relationship between GC content in surrounding noncoding regions (introns and intergenic regions) and the base composition of coding regions (*R^2^* < 0.01, *P *<* *0.1142) within each window. These results indicate that processes shaping global, rather than local, base composition account for GC content and potentially synonymous codon usage in coding sequences (CDSs). Second, we examined the relationship between variation in the local recombination rate inferred from natural sequences and local GC content. We found no evidence for an association between the number of recombination events in a region and the GC content of that region across the whole genome (*R^2^* < 0.0003, *P *<* *0.34; see supplementary fig. S2*A*, Supplementary Material online) or on any of the individual chromosomes (see supplementary fig. S2*B*, Supplementary Material online). Third, we implemented a gross-scale test based on the observation that the proximal half of chromosomes tend to show more crossover events than the distal half (Bloomfield et al. 2019). For this, we used total GC and GC3 (log_10_ transformed) of genes in each region (excluding TEs, which tend to have higher GC and tend to be proximally located) and find no difference between genes located on the proximal or distal halves of chromosomes for total GC (*t*_9563_ = 1.23, *P *=* *0.22) or GC3 (*t*_9563_ = 1.9, *P *=* *0.06). Fourth, we tested whether the proportion of GC in introns explains total GC or GC3 proportion (all log_10_ transformed) in exons and find that intronic GC explains only 0.4% of the variation in GC3 and <0.01% of the variation in total GC. These results indicate that there does not appear to be a strong global process such as gBGC that simultaneously modulates the intronic and exonic GC. Finally, we examined whether the frequency spectrum of AT → GC SNPs differed from the frequency of GC → AT SNPs in introns (see supplementary table S1, Supplementary Material online) and find no difference in any properties of the allele frequency spectrum (two-sample Kolmogorov–Smirnov test, *D *=* *0.04, *P *=* *0.33; see supplementary fig. S3, Supplementary Material online). gBGC would be expected to shift the frequency distribution of AT → GC SNPs above that of GC → AT SNPs, and hence we see no evidence for its influence here.

### Most Variation in Synonymous Codon Use Is Neutral

**Fig. 1.**
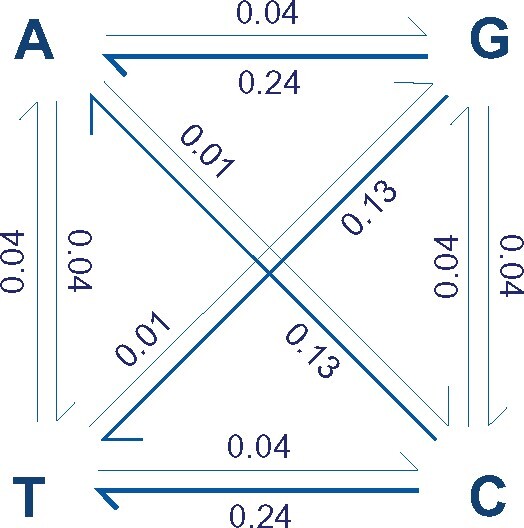
Nucleotide substitution matrix. For each SNP, variants were classified as ancestral (the nucleotide segregating at higher frequency) or derived (the nucleotide segregating at lower frequency). Derived variants were considered mutations from the ancestral allele, and the proportion of all mutations from one nucleotide to each other nucleotide was estimated. Values are proportions of mutations that belong to each category. For example, 1% of all mutations are A to C transversions.

To characterize the expected neutral pattern of codon usage, we modeled the expected distribution of codon frequencies at equilibrium base composition (GC_eq_), which essentially reflects mutation–drift balance. Logically, the relative frequency at which alternative synonymous codons are used will reflect the balance of mutational bias pushing codon use toward that expected at GC_eq_ and selection pushing away from that expected at GC_eq_. Therefore, to characterize the expected neutral pattern of codon use, we first needed to establish the pattern of mutation between nucleotides. Mutation accumulation lines have previously been used to investigate the mutation rate in *D. discoideum* (Saxer et al. 2012). However, the conclusions were drawn from a very small number of mutations and hence provide limited information on the relative rates of different types of mutational change (i.e., the differential mutation rate between the four nucleotides). To overcome this issue, we instead used estimates based on rare segregating variants from regions evolving under neutrality or close to neutrality. SNP data from noncoding regions (intergenic and intronic sites) of 67 *D. discoideum* natural strains (de Oliveira et al. 2019) were used to extract information about the underlying mutational process and compute the nucleotide substitution matrix (fig. 1 and supplementary fig. S4, Supplementary Material online). The estimated substitution matrix is remarkably stable whether it is based on intergenic or intronic SNPs (*R *=* *0.996; see supplementary table S2, Supplementary Material online), and hence, we use the combined estimates. This analysis reveals that transitions (which always change GC class) and interclass mutations (GC → AT and AT → GC) are much more common toward AT than away from it and will thus drive down GC content as mutations arise and drift to fixation.

Genomic GC_eq_ was calculated from the pattern of relative mutation rates (fig. 1) using a system of linear equations (Sueoka 1962). This analysis predicts a GC_eq_ content of approximately 12.2%, which is very close to the value observed in noncoding regions (14%). This suggests that GC content of noncoding regions is close to equilibrium, although the role of selection on some noncoding features (e.g., regulatory sequences) and/or processes like dinucleotide mutation biases presumably result in a slightly higher GC content (compared with the mutational equilibrium). In contrast, GC content in coding regions (∼27.4%) is considerably higher than both GC_eq_ and GC at non-CDSs. This suggests that recurrent selection is opposing the strong mutational bias toward AT accumulation in CDSs. This is perhaps expected since protein function is determined by amino acid composition, and most amino acids (14 of 20) require codons with at least one G/C. This is supported by the fact that relative GC content at position three is lower than that at other codon positions (GC1 = 36%, GC2 = 32%, GC3 = 14%). Indeed, if we remove the two amino acids that have no alternative codon options (methionine and tryptophan, both of which have a third position G), the GC content at position three is 12.2%, which exactly matches the expected GC_eq_ value. Consequently, we see that the overall pattern of codon use is hugely biased toward the AT-richest codons, with most frequently used codon for every amino acid and stop signal always being one of the most AT-rich codons (supplementary table S3 and fig. S5, Supplementary Material online). Hence, it appears that selection on synonymous codon usage does not show an overall bias toward AT or GC ending codons such that their overall use matches GC_eq_.

**Fig. 2.**
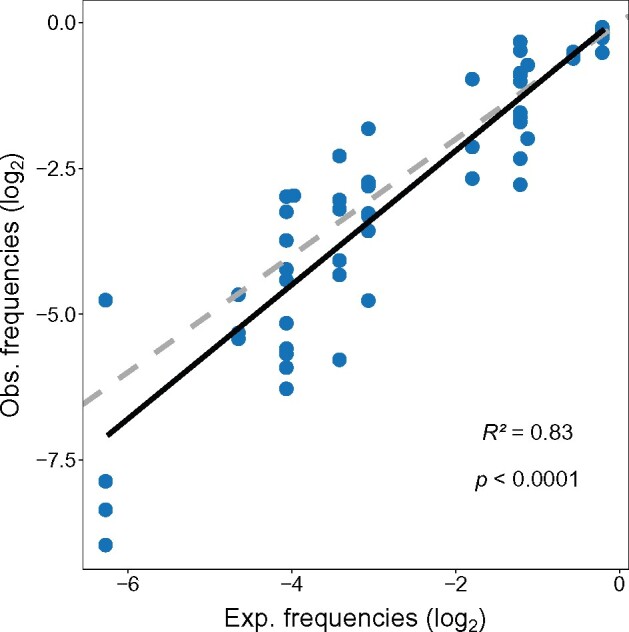
Base composition at mutational equilibrium (GC_eq_) explains most of the variation in the relative frequencies of synonymous codon usage (with both frequencies on a log_2_ scale). The solid line represents the best-fit relationship (from a regression of observed frequencies on expected, where intercept = 0.1 slope = 1.15), whereas the dashed line indicates the 1:1 relationship.

We used the estimated GC_eq_ value to calculate the expected synonymous codon frequencies under neutrality, which assumes that codon use is simply a product of base composition probabilities at each position of a codon. We then rescaled expected relative codon usage to the frequency of the amino acid they encode to obtain relative synonymous codon frequencies excluding stop codons and amino acids encoded by only one codon (methionine and tryptophan). The expected distribution of relative synonymous codon frequencies explains a remarkable 83.3% of the variation in the observed relative codon frequencies (on a log_2_–log_2_ scale *P *<* *0.0001; fig. 2).

The deviations in relative codon usage from the neutral expectation (which represents ca. 17% of the variation in codon use) are assumed to reflect the action of natural selection. However, it is possible that our model of expected codon frequencies at mutational equilibrium is an oversimplification of the neutral expectation. For example, if more complex patterns of dinucleotide or trinucleotide mutations occur, the probability of a given nucleotide triplet could be more than the simple “sum of its parts.” This would be particularly important at repeat-rich DNA sequences, because the repeats increase the chances of polymerase slippage during DNA replication (Ellegren 2004). Therefore, we tested whether trinucleotide combinations, which are commonly found in the *D. discoideum* repeat-rich genome (Eichinger et al. 2005), deviate from the predicted product of expected nucleotide frequencies under mutational equilibrium. The frequency of trinucleotide combinations under neutrality was estimated by counting the number of each in noncoding regions in all three frames. These counts were then treated as pseudocodons to obtain synonymous codon frequencies under neutrality (as these regions are not translated). The synonymous codon frequencies of these neutrally evolving pseudocodons can be largely predicted from the frequencies expected at mutational equilibrium (*R*^2^ = 0.87 on a log–log scale, *P *<* *0.0001), suggesting that mutational processes are mostly acting on a pointwise manner. The deviations of the pseudocodon frequencies from that expected at mutational equilibrium should reflect mutational processes such as biased insertion/deletion events. These processes are presumably responsible for the fact that the relative frequencies of these pseudocodons are far worse at predicting relative codon use (*R*^2^ = 0.74, *P *<* *0.0001) than is the pattern predicted from the independent nucleotide mutation pattern (i.e., the GC_eq_ model; *R*^2^ = 0.83, *P *<* *0.0001). This is perhaps unsurprising because synonymous mutation is essentially constrained to be pointwise, whereas mutations in noncoding regions may be less constrained, allowing insertion/deletion processes to play a role.

These findings suggest that deviations in codon frequencies from the neutral expectation based on base composition equilibrium are caused by selection shaping adaptive codon usage. Hence, codons used more frequently than the neutral expectation are considered evolutionarily “preferred,” whereas those used less often than expected are “unpreferred,” with the magnitude of the deviation from neutrality reflecting the relative strength of selection. This quantitative measure of “adaptive codon preference” is formally captured as a log fold change in the use of codons relative to the neutral expectation, calculated as the difference in the log_2_ relative frequency of a codon observed in the genome from the neutral expectation. This approach therefore provides a quantitative measure of adaptive codon preference that is directly tied to the effects of selection, rather than being inferred from the pattern of codon usage in any class of genes (e.g., codon usage in the most highly expressed genes, which is commonly interpreted as codon preference).

Based on this quantitative measure of adaptive preference, we can investigate the pattern of codon preference to see if there is any obvious structure. Of the 59 codons included in this analysis, 25 show positive deviations from neutrality, that is, are favored by selection (and hence 34 show a negative deviation). Of the 25 preferred codons, we see no obvious bias in terms of the nucleotide present at the third codon position (A = 6, T = 8, G = 3, C = 8). Furthermore, even if we restrict this analysis to the single most preferred codon for each amino acid (i.e., the codon showing the largest positive deviation from neutrality), we do not see any pattern of preference for particular nucleotides (A = 3, T = 6, G = 2, C = 7), with an even split of amino acids having a most preferred codon that is GC and AT ending. This same basic pattern holds at 2-fold amino acids (A = 1, T = 3, G = 2, C = 3) and 4-fold (A = 1, T = 2, G = 0, C = 2).

### Segregating Synonymous Polymorphism Reflects Selection on Codon Preference

**Fig. 3.**
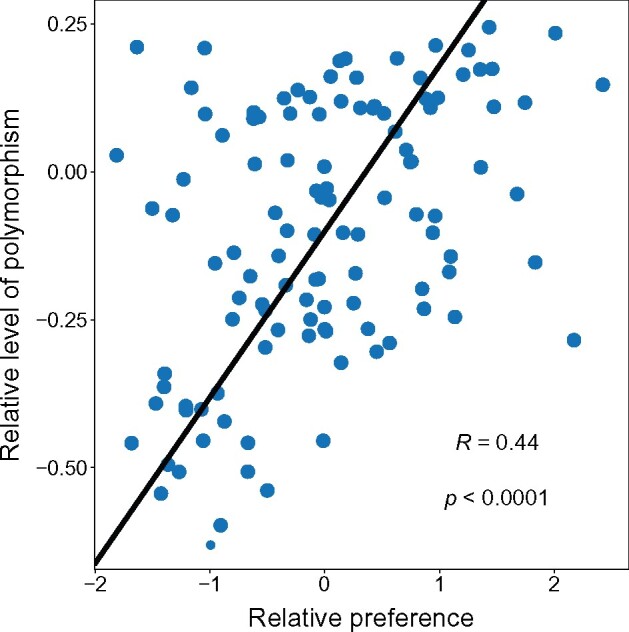
The relative level of polymorphism in each class of synonymous mutation is positively correlated to the relative preference associated with that synonymous change. “The relative level of polymorphism” is the log_10_ difference between the proportion of polymorphism in a mutational class compared with the neutral expectation. The “relative preference” is defined as the difference between the preference of the codon associated with a given synonymous mutation and the average preference of all synonymous mutational options for the given resident codon. Individual points correspond to the different possible synonymous mutational classes. The line represents the best-fit line from a reduced major axis regression model.

If our measure of adaptive codon preference is indeed adaptive, then mutations that lead to less preferred codons should be removed more rapidly by purifying selection than mutations that lead toward more preferred codons (so the impact of a mutation on fitness can be inferred from the change in codon preference it causes). We would therefore expect synonymous SNPs that lead to relatively lower preference codons to be less common than SNPs that lead to relatively higher preference codons (since the SNPs leading to higher preference codons would experience weaker purifying selection or could potentially be favored by positive selection). To test this prediction, we characterized the patterns of naturally occurring levels of synonymous SNP variation associated with each possible type of synonymous codon change (supplementary table S4, Supplementary Material online) using a set of 67 genome sequences (de Oliveira et al. 2019). We then characterized the relative proportion of SNPs associated with each possible type of synonymous mutation (so our analysis is restricted to codons associated with amino acids for which there are more than two alternative codons) and the relative preference of the codons created by those mutations (supplementary table S4, Supplementary Material online). This approach is based on the hypothesis that the proportion of mutational variation within each codon can be predicted based on the expected relative fitness effect of those mutations (so most mutations within a given codon should be in the class that has the highest preference relative to other synonymous possibilities). By modeling variation within each codon, this approach accounts for the variation in the preference of the “resident” codon and focuses on the pattern of preference caused by the mutations (hence it examines the distribution of mutations within codons in relation to the preference of the codons produced by those mutations). We find that there is a relatively large positive correlation between the proportion of polymorphism in a given class of synonymous mutation and the preference of the resulting codon relative to the synonymous alternatives (*R *=* *0.44, *P *<* *0.0001; fig. 3). Therefore, we see a clear signature of purifying selection within codons, with selection removing less of the mutational variation when it is associated with relatively more preferred codons and vice versa. Even for highly preferred codons (for which all synonymous mutations reduce codon preference), this means that SNP variation will tend to be associated with the least “harmful” class of mutation (i.e., with the synonymous codon causing the smallest decrease in preference).

### Conditional Selection Reduces the Magnitude of Codon Preference in Genes

Codons in genes experiencing relaxed selection are expected to show a pattern of codon usage that shifts toward the neutral expectation, which corresponds to lower codon preference (i.e., the pattern predicted at GC_eq_). To test this hypothesis, we calculated gene-level measures based on the preference of the codons within individual genes. This genic level analysis allows us to examine patterns across nearly all genes in the genome at the same time (with the only restriction being that the model can only include genes with available expression data). Using these measures, we tested whether genes expressed only in the developmental part of the life cycle (which are therefore conditionally expressed) show a different level of codon preference compared with genes expressed in all parts of the life cycle. Previous analyses have demonstrated that the conditionally expressed (“sociality”) genes show clear signatures of relaxed selection compared with the nonconditionally expressed (“nonsociality”) genes (de Oliveira et al. 2019), presumably because they are essentially neutral (unexpressed) in most generations. Therefore, we would expect these genes to show a shift toward use of less preferred codons since that corresponds to a shift toward neutrality. Using the gene-level measures of codon preference for the sociality and nonsociality genes, we find that, as expected, sociality genes show a lower average preference (0.070) than nonsociality genes (0.096) (*t*_7551_ = 10.81, *P *<* *0.0001). Hence, we see a clear pattern where genes experiencing relaxed selection show lower preference, which corresponds to a shift toward the neutral expectation.

### Highly Expressed Genes Show Higher Levels of Codon Preference

Our analyses support the conclusion that adaptive codon preference reflects the direction and strength of selection on codons. Many previous studies have assumed that highly expressed genes experience stronger selection, and thus the pattern of CUB in highly expressed genes can be used to infer codon preference. However, it is possible that higher levels of expression impose specific selective constraints such that the pattern of codon use deviates from that expected solely due to a shift toward stronger selection. To address this question, we examined patterns of codon usage in the most highly and lowly expressed genes.

For this, we compiled publicly available transcriptome data in *D. discoideum* (Parikh et al. 2010; Nasser et al. 2013; Rosengarten et al. 2015) and identified the 1,000 most highly and lowly expressed genes (based on the average level of expression throughout the life cycle, excluding the conditionally expressed sociality genes and genes showing zero expression overall). We then counted the total number of times each codon appears in each class of genes and calculated the relative frequency with which each codon is used within each amino acid (following the methods outlined above; see supplementary table S3, Supplementary Material online). For each gene set, we then calculated a measure of preference for each codon that is private to each gene class as the log fold difference between the observed relative codon frequencies within each gene set and the neutral pattern expected at mutational equilibrium (based on the genome-wide estimate of GC_eq_). We also calculated gene-level adaptive codon preference (see above) to compare the average level of codon preference between expression classes.

The private measures of codon preference calculated separately for the highly and lowly expressed genes (i.e., those measuring the deviation in codon use in these classes from the neutral expectation) closely match the genome-wide pattern (*R *=* *0.95 for the highly expressed and *R *=* *0.92 for the lowly expressed genes, *P *<* *0.0001 in both cases; see supplementary table S3, Supplementary Material online). Furthermore, we find that the average size of the deviations from neutrality (measured as the absolute value of the private measures of codon preference in each class) do not differ between lowly expressed genes and the overall genomic pattern (0.34 for all genes and 0.29 lowly expressed genes, *t*_57_ = 1.286, *P *=* *0.21), indicating that not only is the pattern of preference within lowly expressed genes highly correlated to the genome-wide pattern, it is also of a similar magnitude. However, we find that the deviations from neutrality in the highly expressed genes are significantly larger than the genome-wide pattern (0.61 vs. 0.34, *t*_57_ = 3.90, *P *=* *0.0003), indicating that the pattern of deviations from neutrality are similar, but the highly expressed genes are much further from neutrality.

We see a grossly similar pattern by comparing the gene-level average preference of codons used in highly and lowly expressed genes to that of the remaining (intermediate expression) genes. However, although the difference is small, we find that lowly expressed genes show significantly lower mean preference (0.048) than those with an intermediate level of expression (0.082, *t*_7550_ = 17.73, *P *<* *0.0001). In contrast, we see that highly expressed genes show significantly higher mean preference than those with intermediate expression (0.20, *t*_7550_ = 59.6, *P *<* *0.0001). These results indicate that the gross pattern of codon preference in highly and lowly expressed genes mostly mirror the pattern we see across all genes, with the primary differences being the magnitude of the deviations from neutrality and a shift in the central tendency. We also see clear evidence that lowly expressed genes are much more similar to the rest of the genes in the genome than are the most highly expressed genes.

### Adaptive Codon Preference Can Be Inferred from Highly Expressed Genes, but Not Perfectly

**Fig. 4.**
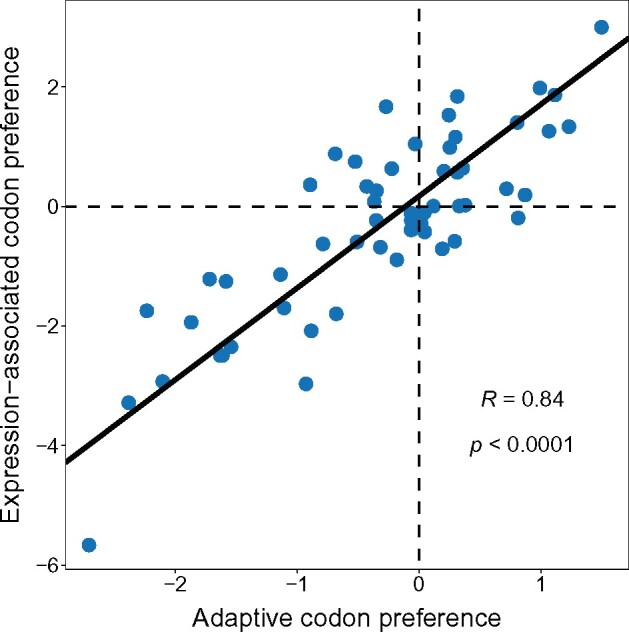
Adaptive codon preference is correlated to, but not the same as, codon preference derived from highly expressed genes. “Expression-associated codon preference” is defined by comparing codon use from the 1,000 most highly expressed genes to the 1,000 most lowly expressed, whereas “adaptive codon preference” represents deviations of relative codon frequencies from the mutational null. The line represents the best fit from a reduced major axis regression model.

Highly expressed genes show a pattern of codon preference that suggests they are under stronger selection. Since most studies base their characterization of codon preference on the pattern of codon use in highly expressed genes, we next examined whether the pattern of CUB associated with gene expression alone (i.e., which is independent of the neutral expectation) could be used to accurately recapitulate the pattern of codon preference derived from our evolutionary model. To test this, we calculated the log_2_ fold difference in the relative use of each codon in the highly expressed class versus the lowly expressed class (using the 1,000 most highly expressed and 1,000 most lowly expressed as above; see supplementary table S3, Supplementary Material online). We find that this “expression-associated codon preference” does indeed approximate the pattern of adaptive codon preference derived from our model (*R *=* *0.84, *P *<* *0.0001; fig. 4). However, the two measures of codon preference are not identical, suggesting that selection on codon usage or differences in mutational processes that are specific to the context of highly expressed genes could affect codon use in relation to expression levels (and potentially lead to misleading inferences). For example, we see a large increase in the use of the AAC codon for asparagine in highly expressed genes compared with lowly expressed genes (hence positive expression-associated preference), but this is a codon that shows negative adaptive codon preference (i.e., is used less frequently than expected under neutrality across all genes). We see a similar shift in the AGC codon for serine, where the expression-associated preference opposes adaptive preference. Interestingly, the nine codons that show positive expression-associated preference, but negative adaptive preference all end in a C or T (C = 6, T = 3). Similarly, we see a number of codons that show a negative expression-associated preference but positive adaptive preference (e.g., the GCA codon for alanine), of which all are A or T ending (*N* = 4 and 3, respectively). This suggests that codons with particular properties may be more likely to show opposing expression-associated and adaptive preferences. Indeed, if we look across all codons, we see a significant pattern (*χ*^2^ = 10.3, *P *=* *0.017) wherein, of the 28 that show higher expression-associated than adaptive preference, 14 are C ending whereas only one is A ending (T = 7, G = 6). Likewise, of the 31 codons showing lower expression-associated preference than adaptive preference, 13 are A ending and only 2 are C ending (T = 9 and G = 7), which is a significant departure from random (*χ*^2^ = 9.26, *P *=* *0.026). These patterns suggests that selection favors C ending codons and disfavors A ending codons in highly expressed genes, which is a pattern that does not appear when we consider deviations from neutrality across all genes, where codons that have a positive adaptive preference value are relatively evenly distributed across classes (A = 6, T = 8, G = 3, C = 8, *χ*^2^ = 1.58, *P* = 0.66). It is also possible that codon use in highly expressed genes is different because they experience a different pattern of mutational input, perhaps mediated by transcription associated repair or mutational processes (and hence we would have a different neutral expectation at mutation–drift balance; see below).

### Deviation of Codon Preference in Highly Expressed Genes Does Not Reflect Differential Mutational Bias

To understand whether the difference in codon usage in highly expressed genes could reflect a difference in the mutational profile, we examined the distribution of intronic SNPs in the set of highly expressed genes (supplementary table S2, Supplementary Material online). Using these SNPs, we calculated GC_eq_ for the 1,000 highly expressed genes and find a value very close to that calculated using all other intronic SNPs (11.8% for highly expressed genes and 12.1% for all other genes). Moreover, the observed number of SNPs per mutational class identified in the introns of highly expressed genes does not significantly deviate from the number predicted based on SNP density in the introns of all other genes (*χ*^2^ = 16.3, *P *=* *0.13). These results provide evidence that suggests that the pattern of expression-associated preference deviates from that of adaptive preference because of systematic differences in selection specific to the context of high expression, rather than a difference in mutation. As a result, measures of preference based solely on expression properties of genes can lead to inferences about codon preference that do not apply perfectly across all classes of genes. Furthermore, it suggests that the mechanisms that underlie selection for preferred codon usage are likely to be complex and multifaceted. Indeed, selection for codon usage has been proposed to be due to transcription, translation, or both (Ikemura 1981; Ikemura 1982; Stoletzki and Eyre-Walker 2007; Plotkin and Kudla 2011; Zhou et al. 2016).

### Codon Preference within Genes Suggests Effects on Ribosome Initiation and Elongation

To understand why selection shapes patterns of adaptive codon preference, we examined how levels of preference change with genomic contexts thought to experience differential selection pressures. For example, codon usage within genes has been shown to affect interactions between the mRNA and ribosomes during translation (Tuller et al. 2010; Bentele et al. 2013). It has been suggested that less “optimal” codons are found in higher frequencies near to the start of CDSs (Allert et al. 2010; Tuller et al. 2010; Bentele et al. 2013; Pechmann and Frydman 2013) in order to facilitate ribosome initiation (via reduced RNA stability) (Kudla et al. 2009) and possibly slow elongation at the start of translation (which can increase accuracy and efficiency). To test this idea, we calculated the frequencies of codons at each codon position in genes, both from the beginning of genes (in the 5′ to 3′ direction; starting after first methionine codon) and at the end of genes (starting with the position prior to the stop codon and moving in the 3′ to 5′ direction). At each position, we measured the average overall codon preference.

**Fig. 5.**
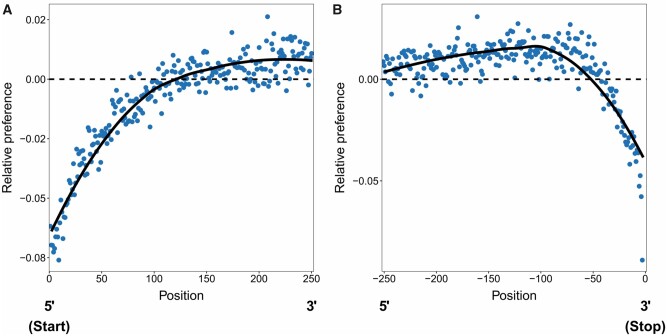
Patterns of codon preference across codon positions within genes. (*A*) the average relative codon preference starting from the beginning of genes, (*B*) and leading up to the stop codon (indicated by position zero, so negative positions are distances before the stop codon). In both plots, the lines represent splines (from an LOESS model), dashed lines represent the genome-wide average preference (zero), and the points represent the individual estimates at each codon position. Relative codon preference represents the average preference of codons present at each codon position relative to (i.e., as a deviation from) the genome-wide average (so a value of zero indicates a match to the genome-wide average, whereas negative values indicate preference below the overall average, etc.).

At the beginning of genes, codon preference starts (going in the 5′ to 3′ direction) below the genome-wide average. Codon preference then increases steadily until it reaches (and then passes) the genome-wide average around codon 120, after which it asymptotes at a value that is higher than the genome-wide mean (see fig. 5*A*). We see a grossly similar pattern at the end of genes, where average codon preference is higher than the genome-wide average until about 50 codons from the stop codon, at which point it rapidly declines approaching the stop codon (in the 5′ to 3′ direction) (fig. 5*B*).

**Fig. 6.**
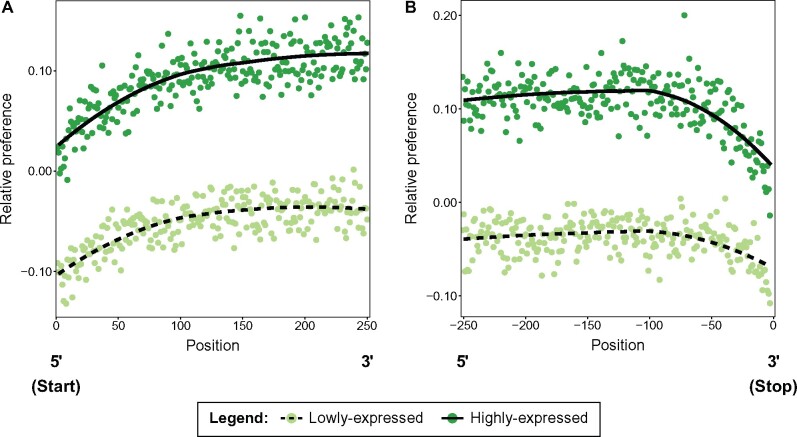
Patterns of codon preference across codon positions within lowly and highly expressed genes. In both plots, the lines represent splines (from an LOESS model; solid line = highly expressed genes, dashed line = lowly expressed genes) and the points represent the individual estimates at each codon position (darker points = highly expressed genes, lighter points = lowly expressed genes). (*A*) Pattern of codon preference starting from the beginning of genes, (*B*) pattern of codon preference leading up to the stop codon (indicated by position zero, so negative positions are distances before the stop codon). Average preference of codons is measured relative to (i.e., as a deviation from) the genome-wide value.

The pattern of codon usage within genes could be taken as support for the idea that selection acts to favor less preferred codon usage or that selection is weaker at the beginning and ends of genes. To distinguish between these possibilities, we first examined patterns of codon usage across codon positions in genes with low or high expression to test whether these patterns change in relation to differences associated with the constraints imposed by the level of expression (and likely the strength of selection). For highly expressed genes, codon preference is above the genome-wide average across all positions (as expected, see above), and we see the same overall pattern in the change in codon preference that is observed across all genes at both the begging and end of genes (fig. 6). For lowly expressed genes, average preference is generally lower than the genome-wide average across all codons and shows the same overall change by codon position that we observe in highly expressed and all genes, though the shift in codon preference is of a lesser magnitude than what we observe in the other classes. These patterns are thus consistent with being driven by selection.

**Fig. 7.**
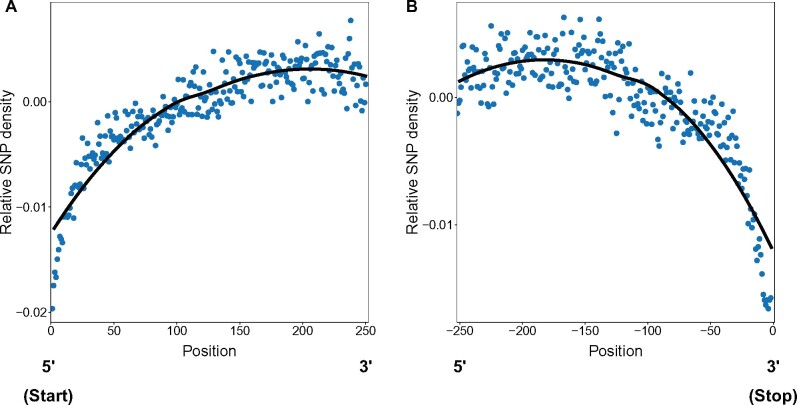
Relative SNP density across codon positions at the beginning and ends of genes. The relative SNP density is the difference between the observed SNP density at a codon position (as a proportion of all SNPs) and that expected based on the expected local mutation rate, which depends on the average GC content at the position.

To further test this idea, we calculated the relative density of synonymous SNP variation across codon positions (adjusted to account for any changes in the proportions of available sites) to examine any signatures consistent with a shift in the balance of mutation and purifying selection, either due to changes in mutation rates or the strength of selection. We see a pattern of SNP density that follows the pattern of change in average preference at both the beginning (fig. 7*A*) and ends of genes (fig. 7*B*). Importantly, the overall shift away from the use of preferred codons near the beginning and ends of genes is what would be expected under either relaxed selection or elevated mutation (because the shift away from use of preferred codons necessarily means a shift toward the pattern expected at mutational equilibrium), but the signature of elevated purifying selection (reflected in reduced segregating synonymous polymorphism) strongly suggests that the shift in codon preference is a consequence of selection on codon usage rather than an enhanced role for mutation and drift. This pattern also implies that codon usage in these regions is experiencing stronger purifying selection than elsewhere in genes given the reduced level of polymorphism.

### Selection on Codon Use Is Partly Explained by tRNA Availability

**Fig. 8.**
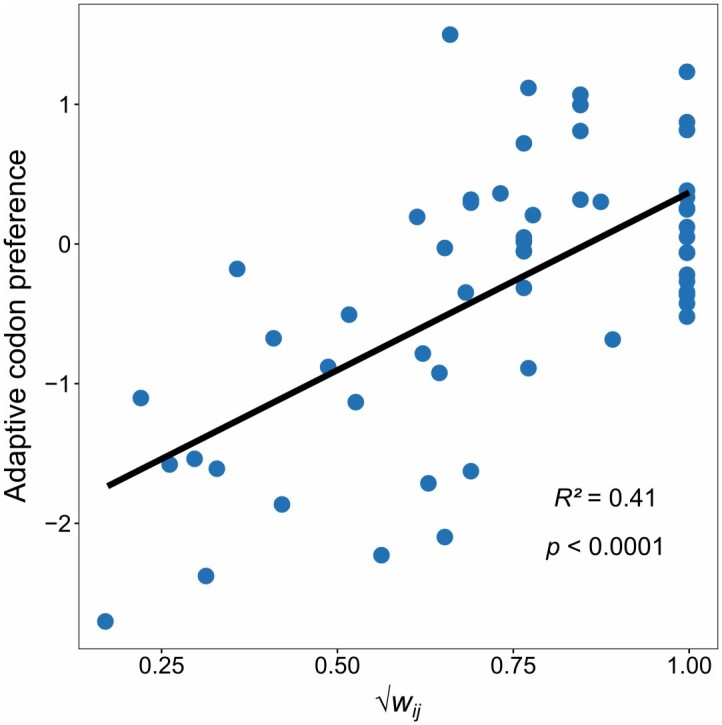
Codon preference is correlated to the relative codon adaptation index (*w_ij_*; after square-root transformation). Codon preference represents the deviation of relative codon frequencies from the neutral expectation at mutational equilibrium.

Selection for codon usage has also been hypothesized to affect translation by favoring codons that match the availability of isoaccepting tRNAs (Ikemura 1981; Gouy and Gautier 1982; Ikemura 1985; Bulmer 1987). To test this idea, we exploited the finding that tRNA gene copy number is strongly and positively correlated to cellular tRNA levels (Duret 2000; Higgs and Ran 2008). To link tRNA levels with patterns of codon use, we derived a new parameter that measures the relative synonymous codon adaptiveness (*w_ij_*). This index is based on dos Reis et al.’s (2003; 2004) codon adaptiveness (*w_i_*). Because our interest is focused on usage of alternative codons encoding the same amino acid (and *w_i_* is defined relative to all codons), *w_ij_* estimates synonymous codon adaptiveness for each codon relative to only the other codons encoding the same amino acid (see Materials and Methods). We find that this relative codon adaptation index (square-root transformed) explains 40.7% (*P *<* *0.0001) of the variation in codon preference (i.e., it explains 40.7% of the variation in codon use that is not explained by the neutral expectation at GC_eq_; fig. 8).

In order to separate the processes shaping use of preferred codons into those that act via the interaction with the tRNA pool and those that appear to be independent of tRNA interactions, we use the relationship between codon preference and relative synonymous codon adaptiveness to partition adaptive codon preference into tRNA-dependent and tRNA-independent components. The tRNA-dependent component, therefore, corresponds to the expected pattern of codon usage given the square-root transformed values of *w_ij_* for a codon and the latter corresponds to the residual from this expectation (controlling for the pattern expected by mutational bias; i.e., it represents the component of codon preference that cannot be predicted from *w_ij_*). As such, the tRNA-dependent component reflects the influence of selection arising from tRNA adaptation on adaptive codon preference, whereas the tRNA-independent component captures other sources of selection.

### Use of Preferred Codons Increases Transcript Stability

Selection can also drive patterns of codon use away from neutrality because it may favor the use of codons because of their effect on mRNA stability, which has been observed in a range of taxa (e.g., *Saccharomyces cerevisiae*, Trotta 2013; Presnyak et al. 2015; *Escherichia coli*, Boël et al. 2016; humans, Hia et al. 2019; Wu et al. 2019), which we refer to as transcriptional selection. For example, such selection could arise because gene expression levels are determined by the rate of mRNA production versus the rate of decay, which can be tied to transcript stability. Since mRNA stability can affect steady-state mRNA levels, a relationship between codon preference and transcript stability could partly explain the observed increase in codon preference observed in highly expressed genes (see above).

To understand the link between codon preference and mRNA stability, we first estimated a measure of stability per site (hereafter referred to simply as “stability”), defined as the global folding Gibb’s free energy (i.e., Δ*G*^0^) divided by CDS length (Lorenz et al. 2011). Transcript stability increases with the average preference of codons in the transcript (*t*_7551_ = 58.4, *P *<* *0.0001, *R*^2^ = 0.311). Both tRNA-dependent and tRNA-independent average codon preference significantly increase transcript stability, but tRNA-independent preference is far more important than tRNA-dependent preference (*t*_7550_ = 72.36, *P *<* *0.0001 for tRNA-independent preference, whereas *t*_7550_ = 20.02, *P *<* *0.0001 for tRNA-dependent preference, corresponding to partial *R*^2^ values of 0.406 and 0.031, respectively). Indeed, by separating total preference into tRNA-dependent and tRNA-independent components, we see that tRNA-independent preference explains far more variation than total preference (*R*^2^ = 0.406 for tRNA-independent preference compared with *R*^2^ = 0.311 for total preference), which suggests an adaptive role for tRNA-independent preference associated with transcript stability that is unrelated to adaptation to the available tRNA pool. This finding suggests that selection to optimize expression by usage of differential codons is, at least in part, achieved through increased transcript stability. Since this increased stability is primarily a result of tRNA-independent preference, this relationship provides a hypothesis for why selection favors tRNA-independent preference.

### Sources of Selection Driving Codon Preference Change across Codon Positions within Genes

Given that there are relationships between preferred codon usage and tRNA availability and the position within genes, we next examined the relationship between these phenomena. For this, we measured the average relative tRNA-dependent and tRNA-independent preferences at each codon position within genes.

**Fig. 9.**
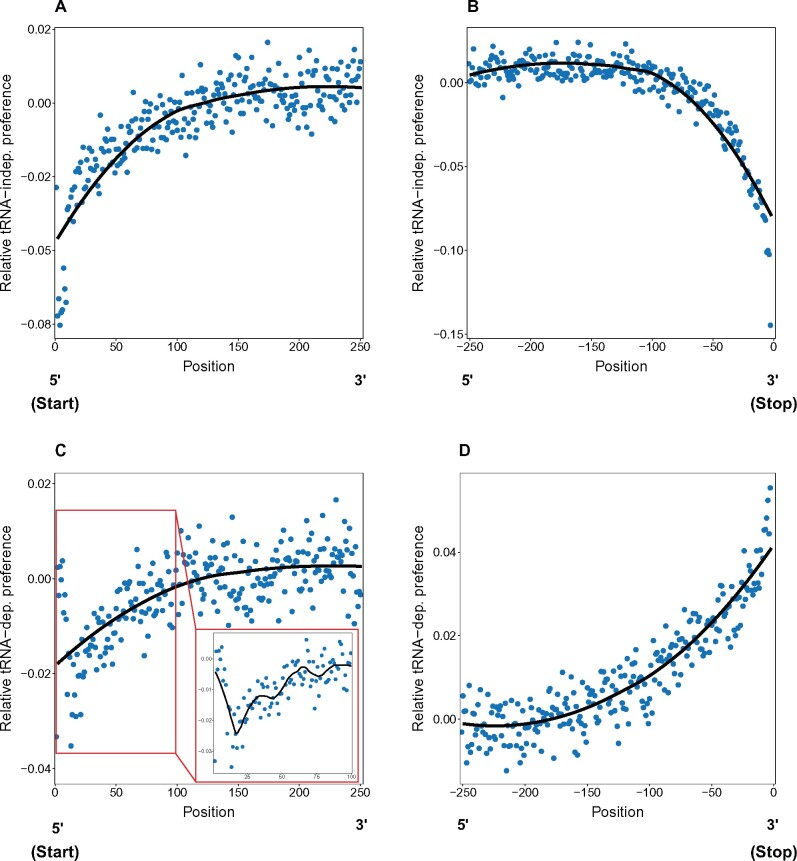
Patterns of average relative tRNA-dependent and tRNA-independent codon preference across codon positions within genes. The first two panels (*A* and *B*) show patterns of tRNA-independent preference at the beginning (*A*) and the ends of genes (*B*). The other two panels (*C* and *D*) show patterns of tRNA-dependent preference at the beginning (*C*) and ends (*D*) of genes. At the beginning of genes, codon positions are numbered from the start codon, whereas at the ends of genes, the negative positions give the distance before the stop codon. In all plots, the lines represent splines (from an LOESS model), and the points represent the individual estimates at each codon position. All preference values are given as deviations from the genome-wide mean for that given type of preference (so the zero position indicates values that match the overall mean).

At the beginning (going in the 5′ to 3′ direction) and ends (in the 5′ to 3′ direction) of genes the mean tRNA-independent preference shows the same pattern as overall adaptive preference (fig. 9*A*, compared with figs. 5*A* and 9*B* compared with fig. 5*B*). For tRNA-dependent preference, at the beginning of genes, we see a rapid decline over the first ca. 20 codons, and then a steady increase that matches the pattern overall (and of the tRNA-independent preference) (fig. 9*C*). However, at the ends of genes, we see a drastically different pattern in the tRNA-dependent preference (fig. 9*D*), which steadily increases starting about 170 codons from the stop codon and continues to increase until the stop codon. This finding is consistent with the expectation that selection favors use of codons that increase translation rates at the end of genes to facilitate release from the ribosome (Allert et al. 2010; Tuller et al. 2010; Bentele et al. 2013; Pechmann and Frydman 2013). Thus, by dividing codon preference into tRNA dependent and tRNA-independent components, we can see distinct patterns of selection driving codon use away from neutrality, especially the fact that the ends of genes appear to show a particularly strong signature of selection driven by tRNA adaptation.

## Discussion

The AT-biased genome of the social amoeba *D. discoideum*, coupled with estimated patterns of mutation, and an absence of gBGC, has allowed us to define codon preference in evolutionary terms. It therefore can be described as a property of the genome that reflects the degree to which natural selection has favored the use of some codons, driving their frequency away from neutrality. For example, even though we see that the overall pattern of codon usage is strongly biased towards the use of the AT-richer codon alternatives (Sharp and Devine 1989; Eichinger et al. 2005), this does not mean that these codons are evolutionarily preferred since this bias is expected under neutrality (due to background mutational processes). To identify when codons are used more or less frequently than the neutral expectation (which reflects mutation–drift balance), we modeled the neutral expectation of codon frequencies at base composition equilibrium (GC_eq_) using patterns of SNP variation (fig. 1). A large majority (83%) of synonymous CUB can be explained solely by mutational biases toward AT accumulation in this genome (fig. 2). However, the remaining variation (ca. 17%) shows signatures of natural selection shaping synonymous codon usage (signatures of purifying selection, dependence on level of gene expression, etc.) in order to influence properties associated with transcription and translation. Therefore, we refer to this variation as adaptive codon preference to reflect the potential adaptive value of the pattern.

In many analyses, codon preference is inferred solely based on comparison of codon use in highly versus lowly expressed genes (i.e., independent of the neutral expectation). This mode of analysis comes with assumptions, such as the mutation and nonselective fixation processes are no different between gene classes. It can also be tautologous—lowly expressed genes are deemed less adapted when their CUB is not as skewed in the direction of the optimal codon set, even if for such genes selection may not be favoring “optimal” codon usage. Because codon preference appears stable across different classes of genes, differing primarily in the magnitude, not in overall pattern, we were able to examine whether the pattern of adaptive preferred codon use matches codon preference inferred from gene expression. This indicates that in the absence of a neutral expectation, an approach tied to an analysis of gene expression variation provides a reasonable estimate of adaptive codon preference. However, we find evidence that some of the shift in codon use in highly expressed genes appears to be shaped by selection that is specific to the context of high expression, rather than reflecting an overall selective advantage to the use of certain codons more broadly. At the extreme, this difference is manifested by an increased use of certain codons in highly expressed genes that are used less often than the neutral expectation across the genome overall. This finding suggests that some caution is therefore needed when basing inferences purely on the assumption that codon use in highly expressed genes entirely captures the evolutionarily relevant pattern of codon preference across the genome.

The pattern of selection reflected in codon preference is consistent with roles in the optimization of protein translation (fig. 8). A large fraction (ca. 41%) of adaptive codon preference can be explained by the relative availability of isoaccepting tRNAs for the alternative codons within each amino acid. Codon preference also impacts translation through its effect on mRNA stability, which increases with the average preference of the codons within a gene. Stability increases mRNA steady-state levels and has been reported as an important mechanism of optimization of expression (Kudla et al. 2006; Trotta 2013). Interestingly, the effect of the use of preferred codons on transcript stability is almost entirely due to the component of preference that is independent of the relative tRNA adaptiveness of the codons. Therefore, the connection between codon use and transcript stability provides a source of selection shaping codon preference that would not be apparent from an analysis focused solely on adaptation to the tRNA pool.

Our analyses also allow more subtle effects of selection on codon usage to be detected. For example, we find that patterns of codon preference shift across codon positions in genes, generating an intragenic “codon landscape,” with preference lower toward the start codon and approaching the stop codon of genes (fig. 5). The pattern of reduced codon preference near the start and end of genes is consistent with reduced strength of selection or increased mutation, given that a reduction in codon preference corresponds to a shift in codon usage toward the neutral (mutation–drift) expectation. However, the reduction in frequency of preferred codons near gene boundaries appears to be adaptive. Shifts in the use of preferred codons are stronger among highly expressed genes (fig. 6), consistent with highly expressed genes being under stronger optimizing selection to counteract the influence of mutation. Furthermore, we see less synonymous polymorphism approaching the start codon (in the 3′ to 5′ direction) and the stop codon (in the 5′ to 3′ direction) across all genes (fig. 7), which also strongly suggests that those regions are experiencing elevated purifying selection, rather than relaxed selection or elevated mutation. These findings are thus consistent with the idea that shifts in codon use at the 5′ and 3′ ends of genes influences the temporal and spatial nature of interactions between mRNA transcripts with ribosomes (Quax et al. 2015). In the region near the start codon, selection for less optimal codons may slow down transcriptional elongation at the beginning of genes to increase accuracy and ensure more even spacing of protein synthesis at ribosomes, which reduces ribosomal “traffic jams” (Quax et al. 2015). The scale of this “ramp” may be dictated by the length of the exit tunnel of the ribosome (Quax et al. 2015), which is approximately the scale over which we see the pattern of codon preference shift near the start of CDSs (Fredrick and Ibba 2010). There is also evidence that codon use near the start codon may reflect selection for reduced mRNA folding, which facilitates initiation of translation (de Smit and van Duin 2003; Kudla et al. 2006; Studer and Joseph 2006; Bentele et al. 2013; Goodman et al. 2013). Although we do not have any estimates of the influence of codons on mRNA folding in our study, it is possible that this phenomenon is related to the influence of preferred codons on transcript stability, with both potentially reflecting binding properties of transcripts.

Taken together, our findings suggest that adaptive preferred codon usage can be measured in *D. discoideum* as a deviation from the neutral pattern. This reveals that selection favoring evolutionarily preferred codons confers a selective advantage through the optimization of gene expression. We suggest that the term “codon preference” should be reserved for departures of codon usage from the neutral expectation (presumably driven by mutational processes) caused by an active role of selection arising from differences in codon fitness.

## Materials and Methods

### Synonymous Codon Frequencies and GC Distribution across the Genome

Relative synonymous codon frequencies (i.e., relative to each amino acid) were estimated from the reference genome (Eichinger et al. 2005) downloaded from Ensembl (Aken et al. 2016; Kersey et al. 2016) and using the R package seqinR (Charif and Lobry 2007). Before computing this codon table, we excluded all non-protein-coding sequences, genes from the mitochondrial genome and from a duplication in chromosome 2, present only in the strain AX4 (reference genome, Eichinger et al. 2005). This censoring was necessary because the codon unity is meaningful only when translated into amino acids, and because genes in these other regions (mitochondrion and duplication) can evolve under different dynamics in comparison with the rest of the genome.

GC content was computed in coding and noncoding regions of all six chromosomes of *D. discoideum*, in windows of 50 kb. In a first sliding-window analysis, windows were separated by step sizes of 1 kb in order to capture patterns of GC levels across chromosomes. In a second analysis, GC was computed in 50 kb windows without overlaps, to compare local GC content in coding and surrounding noncoding regions within each window. In both cases, we used coordinates from Ensembl Protist gene annotation release 46 (Aken et al. 2016; Kersey et al. 2016) to characterize chromosome regions as coding or noncoding DNA (intronic and intergenic). We also used these coordinates to localize a list of genes annotated as TEs in Dictybase (Fey et al. 2013) to test the hypothesis that peaks of elevated GC could be associated to the presence of such elements. Peaks of both lower and higher GC (<5th and >95th percentiles of GC distribution in the 50 kb windows) were identified from noncoding regions, under the assumption that noncoding DNA evolve close no neutrality (whereas base composition could be potentially under selection in CDSs).

Per gene total GC and GC3 content for the coding regions of each gene, as well as the overall GC content of their corresponding intronic regions were also calculated based on gene and CDSs coordinates from Ensemb Protist. Per gene total GC, GC3, and intronic GC were then log_10_ transformed. To reduce noise in the comparisons between these estimates for different classes, short genes (length < 500 bp), TEs, and genes showing conditional expression (de Oliveira et al. 2019) were excluded from these analyses.

### Nucleotide Substitution Matrix and GC_eq_

Overall nucleotide composition is mostly a result of mutational biases (Sueoka 1962; but see also Rocha and Feil 2010), so understanding the evolution of such an AT-biased genome as in *D. discoideum* must include a detailed investigation of mutational processes. Because experimental work on mutational patterns in this system resulted in conclusions drawn from a single SNP (Saxer et al. 2012), we used information from segregating variation to derive general patterns. This data set includes 67 natural strains, and details on the geographical distribution of the strains, sequencing reports, mapping, and SNP calling are provided elsewhere (de Oliveira et al. 2019). Briefly, reads were cleaned for adapters and quality trimmed using Trimmomatic (Bolger et al. 2014). Reads derived from possible contaminants were excluded by binning and simultaneously mapping them to the reference genome of *D. discoideum*, *Paraburkholderia xenovorans lb400*, *Burkholderia ubonensis*, *Paraburkholderia fungorum*, and *Klebsiella pneumoniae*; and assigning them according to the best mapping score using BBSplit, part of the BBMap package (version 36.27) (Bushnell 2016). Reads binned with *D. discoideum* or not mapped to any of the bacterial genomes aforementioned were pooled together and mapped to the *D. discoideum* reference genome using NextGenMap (Sedlazeck et al. 2013). SNP calling was then performed using Genome Analysis Toolkit (McKenna et al. 2010). Resulting SNPs were filtered with a static threshold for quality by depth (QD) < 2.0, an FS score (Phred-scaled probability that there is strand bias at the site, based on a Fisher exact test) > 60.0 and a root mean square mapping quality (MQ) over all the reads at the site of < 30.0. Any strain with a missing call rate higher than 0.3, any site called in <90% of the remaining strains (i.e., in < 60 out of 67 strains), and multiallelic site or indel were also removed. This resulted in a data set of 279,807 SNPs across 67 strains.

In order to calculate an estimate of the expected GC under equilibrium, SNPs were filtered to include only those from noncoding regions, since these are expected to be mostly governed by mutation–drift balance. Directionality was inferred from polarization of rare alleles in comparison with the common alleles. Relative rates of mutation between different nucleotides were first calculated by scaling SNP counts to the number of sites and then rescaled again by dividing these per-site measures by their sum to get a relative rate (such that the sum of the rates equals 1). This information was used to generate the nucleotide substitution matrix with proportion of substitutions in all directions of mutational space, which in turn was used to derive the expected GC under equilibrium using Sueoka’s (1962) equation:

(1) $${\mathrm{GC}}_{\mathrm{eq}}=\frac{\left(\mathrm{AT}\;\to \;\mathrm{GC}\right)}{\left(\mathrm{AT}\;\to \;\mathrm{GC}\right)+\left(\mathrm{GC}\;\to \;\mathrm{AT}\right)}.$$

This same analysis was carried out for a set restricted to SNPs in the introns of the 1,000 most highly expressed genes (see below for the definition) to generate a separate estimate of the mutation profile and GC_eq_ for this category of genes. Again, intronic coordinates were based on the Ensembl Protist gene annotation release 46 (Howe et al. 2020). The pattern of mutations per site calculated for all genes was used to generate an expected distribution for intronic mutations (based on the number of different classes of available sites in the introns), which was normalized to account for a difference in the absolute level of polymorphism in the introns of highly expressed genes compared with all noncoding sites across the genome (with the introns containing about 34% less SNP variation). For this analysis, we combined the equivalent mutational classes because SNPs cannot be assigned to strands (e.g., A to C and T to G SNPs are treated as a single class). We then compared the observed number of mutations in each class within the introns of highly expressed genes to those expected based on the genome-wide mutational profile of noncoding sites using a chi-square test with five degrees of freedom (reflecting the six classes of mutational change).

### Estimation of Rates of Recombination

To assess local variation in recombination rates and its relationship with local GC content, we estimated population-scaled recombination rates (Rho, ρ) for our sample of strains (de Oliveira et al. 2019) along nonoverlapping sliding windows of 5 kb in length, covering the whole genome, using a machine-learning approach implemented in the FastEPRR R package (Gao et al. 2016). To estimate confidence intervals for ρ for each window, we ran 1,000 replicates, using default ρ values for simulating the training sets. Afterward, 1 × 10^−6^ was added to the estimated value of ρ of each window, to allow for windows with an estimate of ρ = 0 to be included in subsequent analysis, and estimates of ρ where then log-transformed. We furthermore calculated the overall GC percentage, over the same genomic windows, using the Biostrings R package (version 2.52) (Pagès et al. 2020). We then measured the correlation between the log-transformed recombination rate estimates and the overall GC percentages in the genomic windows. This was done for all windows across the genome as well as separately for each chromosome.

### Expected Relative Frequencies and Identification of Preferred Synonymous Codons

The estimated value of GC_eq_ (∼12.16%) from equation (1) was first used to calculate the expected absolute codon frequencies from the product of the expected frequencies of the component nucleotides. For example, the expected frequencies of the two codons for lysine are: $E[f\left(\mathrm{AAA}\right)]=E[f({A)]}^{3}$, and $E[f\left(\mathrm{AAG}\right)]=E[f{\left(A)\right]}^{2}\times E[f\left(G\right)]$, where the individual nucleotide frequencies are those expected at GC_eq_. The expected frequency of the amino acid is, therefore, the sum of the absolute codon frequencies for all codons coding for that amino acid. For lysine, this would be as follows:

(2) $$E\left[f\left(\mathrm{Lys}\right)\right]=E\left[f\left(\mathrm{AAA}\right)\right]+E\left[f\left(\mathrm{AAG}\right)\right].$$

The total expected frequency for each amino acid was then used to rescale the frequency of the codons within the amino acid to produce the relative frequencies of the codons. For example, the expected relative frequency of the AAA codon within lysine would be:

(3) $$E\left[\hat{f}\left(\mathrm{AAA}\right)\right]=\frac{E\left[f\left(\mathrm{AAA}\right)\right]}{E\left[f\left(\mathrm{Lys}\right)\right]}.$$

The alternative method of estimating expected relative frequencies by computing the emergence of triplets (pseudocodons) under neutrality was performed as follows. Noncoding regions of all six chromosomes were concatenated in a single linear sequence, which was divided in triplets on the three frames. Absolute frequencies of these pseudocodons were calculated based on the sum of counts of each pseudocodon in all three frames. These frequencies were translated into relative frequencies following the methods above, treating the pseudocodons the same way we treated the real codons (i.e., as if they were from CDSs).

To estimate the pattern of preference for different codons, we accounted for the expected frequency of codons driven by base composition bias by subtracting the relative synonymous codon frequencies expected at GC_eq_ (on a log_2_ scale) from the observed relative synonymous codon frequencies (also on a log_2_ scale). This method assumes that codons used more often than predicted under neutrality (Obs_f_ > Exp_f_) must confer an advantage and are favored by selection. Conversely, codons used less frequently than expected by neutral evolution (Obs_f_ < Exp_f_) are assumed to confer a disadvantage and are therefore unpreferred/avoided. The resulting measure of codon preference can be interpreted as a log fold change from neutrality, which is assumed to be caused by selection.

### Association between Codon Preference and Segregating Synonymous Polymorphism

To identify synonymous mutations, we used the Ensembl variant effect predictor (McLaren et al. 2016) to characterize the consequences of SNPs based on the EnsemblProtist gene annotation release 46 (Howe et al. 2020). We tested our hypothesis about codon preference by examining the relationship between the properties of codons and the inferred fitness effects of different types of synonymous mutations. For this, we assume that signatures of selection on synonymous codon usage are captured in the levels of synonymous polymorphism. This is based on the assumption that codon preference imposes purifying selection, such that segregating polymorphism primarily represents deleterious mutational variation. To understand these relationships, we modeled the expected neutral distribution of synonymous polymorphism based on the pattern of mutation estimated from noncoding regions. The expected patterns of mutational variation was compared with naturally occurring patterns measured from a set of 67 genome sequences (de Oliveira et al. 2019).

We examined the correlation between the relative amount of synonymous polymorphism and the change in preference caused by the change in codon associated with each synonymous mutation. To calculate the expected frequency of each synonymous mutation, we then multiplied the genome-wide frequency of a given codon (i.e., the frequencies of the mutational targets, *f_i_*) by the relative proportion of all mutations (*m_j_*) that are in that class of mutation. For example, consider a GCA to GCC change (which represents a synonymous mutation for alanine). The GCA codon has a frequency (*f_i_*) of 0.0164 and the change requires an A to C mutation, which represents a proportion (*m_j_*) of 0.01 of all mutations (see fig. 1), making the raw proportion for this change *p_ij_* = *f_i_m_j_* = 0.0164 × 0.01. These raw values (*p_ij_*) were normalized by dividing by their sum such that each value gives the proportion of all SNPs observed that are expected to be in that given class as follows:

(4) $${\hat{p}}_{ij}=\frac{{f_{i}m}_{j}}{\sum {f_{i}m}_{j}}=\frac{p_{ij}}{\sum p_{ij}}.$$

These proportions are given in supplementary table S4, Supplementary Material online. Using the expected relative proportions of SNPs (eq. 4), we then tested whether the proportion of synonymous mutations within each codon (relative to that expected) is associated with the relative preference of those mutations. This approach controls for variation in the relative selective impact of synonymous mutations across different codons, essentially asking whether the probability of observing different types of synonymous changes from a given codon depends on the preference of the destination codon. For three classes of synonymous mutation, all being very rare codons for arginine (GCA → GCG, CGC → CGG, CGG → CGC), we found no synonymous SNPs. We replaced the zeros with a count of one to allow them to be included in this analysis, but this change makes no difference to the result (*R *=* *0.446 when they are removed, and *R* = 0.440 when they are included).

This analysis is necessarily restricted to codons for which there is more than a single class of synonymous mutation since it relies on there being alternative mutational “options.” Therefore, this analysis is restricted to 114 of the full set of 134 possible synonymous mutations (with the eight mutations being at the first codon position and the remainder at the third position). For each codon, the expected proportion of each of the possible synonymous mutations was calculated from the relative mutation rate. For example, for the GCA codon coding for alanine, there are three possible synonymous mutations resulting in the GCC, GCG, and GCT codons, respectively. The mutation rate for each of these classes of mutation (i.e., for the A to C, A to G, and A to T changes leading to each of these three codons) are approximately 0.01, 0.04, and 0.04. Therefore, we expect to see the three types of synonymous mutations (leading to GCC, GCG, and GCT) in the proportions about 0.11, 0.44, and 0.45. These expected proportions were then compared with the observed proportions (given in supplementary table S4, Supplementary Material online) by calculating the log_10_ of the observed proportion divided by the expected, which provides a deviation measure that was calculated across all possible codons. For example, in the case of the GCA codon, we observed the three classes of synonymous mutations (GCC, GCG, and GCT) in the proportions 0.16, 0.43, and 0.41, resulting in log_10_ frequency deviations of 0.17, −0.01, and −0.04. This result suggests an overabundance of mutations from GCA to GCC and a possible underabundance to GCG and GCT. To understand whether the deviations in mutation proportions per codon reflect selection arising from codon preference, we tested whether the relative preference of each codon predicts the relative abundance of that class of mutation. To be able to analyze patterns across all codons, we scaled the preference values of possible alternative codons by measuring them as deviations from the average preference of those particular codons. For example, in the case of mutations at the GCA codon for alanine, the three possible synonymous mutations (leading to GCC, GCG, and GCT) have preference values of ca. 0.82, −1.62, and −0.41 (compared with the preference of GCA itself, which is 0.31), with a mean of ca. −0.40. These raw preference values were scaled as deviations from the mean, giving them relative preference values of ca. 1.22, −1.21 and −0.01.

### Codon Use in Highly and Lowly Expressed Genes

We used publicly available transcriptome data from vegetative and developmental cycles in *D. discoideum* (Parikh et al. 2010; Nasser et al. 2013; Rosengarten et al. 2015) to identify sets of highly and lowly expressed genes. Expression levels were defined as the average expression after normalization of vegetative and developmental RNAseq libraries (Parikh et al. 2010; Nasser et al. 2013; Rosengarten et al. 2015). Details of the analysis are provided by de Oliveira et al. (2019) and only briefly outlined here. Libraries were normalized using the TMM method (Robinson and Oshlack 2010) implemented in edgeR (Robinson et al. 2010), after removing genes with low counts, following authors’ specifications. Very short (≤500 bp, corresponding to 2,647 genes) and very long genes (the top 2,647 longest genes) were removed from our analyses, and the two classes of top 1,000 highest and 1,000 lowest expressed genes were selected among nonconditionally expressed genes (i.e., genes whose expression is not restricted solely to the social phase). This allows us to have a measure of expression that applies throughout the life cycle and removes the potentially complicating effects of conditionality (see de Oliveira et al. 2019 and below for details on how conditionally expressed genes were identified). We then calculated the frequencies of codon use in each of these two sets of genes and used these frequencies to calculate the average levels of codon preference for each set. We also used the log_2_ transformed relative frequencies to generate a measure of codon preference that is analogous to the one based on the deviation of observed relative frequencies from the neutral expectation (here calculated as the value in highly expressed genes minus the value in lowly expressed genes).

### Parameters of Translational and Transcriptional Selection

Coevolution of codons with the pool of isoaccepting tRNAs is likely to be an important process shaping codon usage. This coevolution hypothesis is often tested by estimating the relative codon adaptiveness (*w_i_*) (dos Reis et al. 2003; dos Reis et al. 2004), which gives a measure of fitness assigned to each codon. A limitation of this method is that *w_i_* is defined relatively to the maximum adaptiveness value across all codons (*W*_max_), including codons for different amino acids. This means that it may not be an appropriate measure for understanding the usage of alternative codons, since different indices of tRNA adaptiveness may be due to differences in amino acid usage rather than differences on the strength of selection on synonymous codons to optimize expression. Therefore, to generate an index that is appropriate for the study of synonymous codons, we rescaled the measure of codon adaptiveness such that it measures the relative values for different synonymous codons for each individual amino acid. Thus, the relative synonymous codon adaptiveness of a particular codon (*w_ij_*) is defined as follows:

(5) $$w_{ij}=\frac{W_{i}}{W_{j\max}},$$

where *W_i_* is the absolute codon adaptiveness (tRNA gene copy numbers after accounting for wobble pairings), and *W_j_*_max_ is the maximum absolute adaptiveness among codons of amino acid *j*. This parameter (*w_ij_*) was estimated by adapting R scripts from dos Reis et al (2003; 2004), after removing codons for methionine, tryptophan (both with a single codon) and stop signal. Following dos Reis et al (2004), we replaced the measure of *w_i_* with the geometric mean of all codons (prior to calculating *w_ij_*) with a value of *w_i_* > 0 for the two codons, where *w_i_* ∼ 0 (the GCA and GCG codons for alanine), which prevents these very low values from driving patterns.

To understand the extent to which codon preference is explained by coevolution with the tRNA pool or by other parameters, we partitioned the variation in codon preference in tRNA-dependent and tRNA-independent components. The tRNA-dependent component corresponds to the component of codon preference that can be predicted based on *w_ij_*, whereas the residual value of codon preference (i.e., the deviation from the preference value expected based on *w_i_*) gives the tRNA-independent component of codon preference.

As a measure of transcriptional selection, we estimated levels of transcript stability based on Gibbs free energy (Δ*G*^0^), using the RNAfold tool (which calculates the global folding of a sequence) from ViennaRNA package (Lorenz et al. 2011). Given the same transcript length, transcripts with lower (more negative) Δ*G*^0^ are more stable. Because transcript length can also influence stability levels (longer transcripts tend to be more stable), we weighted the original parameter by CDS length to obtain an estimate per site.

### Modelling Codon Use across Codon Positions within Genes

To understand whether the overall model of codon use varies across genes, we used a data set filtered for gene length (where very long and very short genes were removed; see above) and calculated the frequency of each possible codon across all genes at each codon position within the genes in both the 5′ to 3′ and the 3′ to 5′ directions (meaning genes were either aligned to the start codon or stop codon before frequencies were calculated). To avoid overlap between the analysis from the 5′ to 3′ and in the 3′ to 5′ directions, we restricted our analysis to the first or last 250 codon positions (depending on the direction). The position-specific table of codon frequencies was used to calculate the average preference (including total preference, and the tRNA-dependent and tRNA-independent components) of codons by simply multiplying the vector of frequencies of the codons at each position and the vectors of codon preference (i.e., the total preference and tRNA-dependent and tRNA-independent components, which were all estimated using the overall genome-wide frequency of codon use).

### Codon Use in Conditionally Expressed Genes

Conditionality of the social cycle has been shown to have an important impact on evolution of the genes expressed only in the social cycle because it dilutes selection, resulting in the Red King process (de Oliveira et al. 2019). Therefore, we contrasted patterns of codon use in conditionally expressed “sociality” genes (which are expressed only in the social phase of the life cycle) with the “nonsociality” genes (which are expressed throughout the life cycle) as defined in de Oliveira et al. (2019) by comparing the means of average preference in the two groups, after removing genes <500 bp.

## Supplementary Material

Supplementary data are available at *Molecular Biology and Evolution* online.

## Supplementary Material

msab099_Supplementary_Data
