# Supplementary material for: Inferring Adaptive Codon Preference to Understand Sources of Selection Shaping Codon Usage Bias
Source: Mol Biol Evol. 2021 Apr 19;38(8):3247–66. doi: 10.1093/molbev/msab099 (PMC8321536; doi:10.1093/molbev/msab099)
Supplement: msab099_Supplementary_Data [file msab099_supplementary_data.zip › Supplementary material.pdf]

Supplementary material

Table S1 (Excel file). Frequencies of intronic SNPs in different SNP categories (in terms of whether the change is AT→AT, AT→GC, GC→AT, or GC→GC). For each SNP we give the genomic position (chromosome and bp position), the resident and derived allele SNPs, the derived allele frequency, and the SNP class.

Table S2 (Excel file). Number of SNPs and available sites for intergenic regions, introns, and the introns of the 1000 most highly expressed genes

Table S3 (Excel file). Codon parameters from *D. discoideum* genome. The table includes counts of codons in all genes and in the 1000 most highly and lowly expressed genes, along with their absolute and relative (within amino acid) frequencies, expected frequencies based on pseudo-codon (triplet) frequencies and at GC_{eq}, the number of tRNAs and the relative tRNA adaptation index (w_{ij}), measures of codon preference in all genes and in the 1000 most highly and lowly expressed genes, and expression-associated preference (measured as the $\log_2$ difference in relative codon use in the highly and lowly expressed genes).

Table S4 (Excel file). Pattern of synonymous SNP variation for all codons that have more than one synonymous alternative. The table includes the total count of SNPs, their expected number (based on the estimated mutation matrix), expected proportion within each codon, the $\log_2$ difference in the observed and expected proportions within each codon, these same values calculated for a set where all cases with zero SNPs were replaced with a single SNP (labelled ‘no zeros’), the preference of the ‘resident’ and ‘mutant’ codon, the average preference of all possible mutations within that codon, and the relative preference of each mutant codon as a deviation from the average of all mutant codon options.

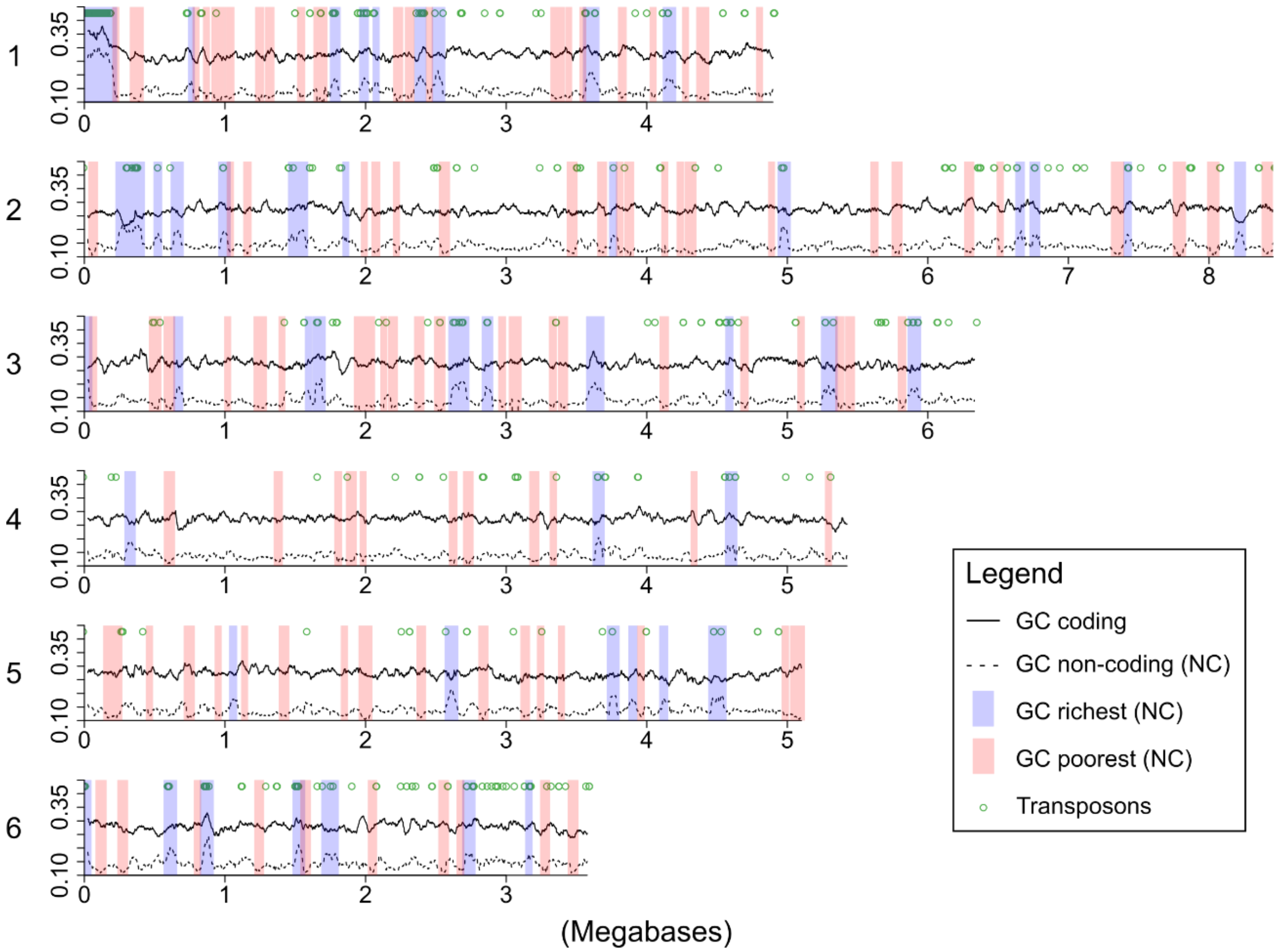

Figure S1: Sliding window analysis of genomic GC content. GC content was estimated for coding (solid lines) and non-coding (dashed lines) sequences in 50Kb windows in 1Kb step sizes across all six chromosomes of *D. discoideum*. Regions with lowest (< 5th percentile) and highest (> 95th percentile) GC contents in non-coding sequences are highlighted in red and blue bars, respectively. Peaks of greatest GC (mostly on chromosomes 1 and 6) are associated with an overrepresentation of transposable elements (green dots).

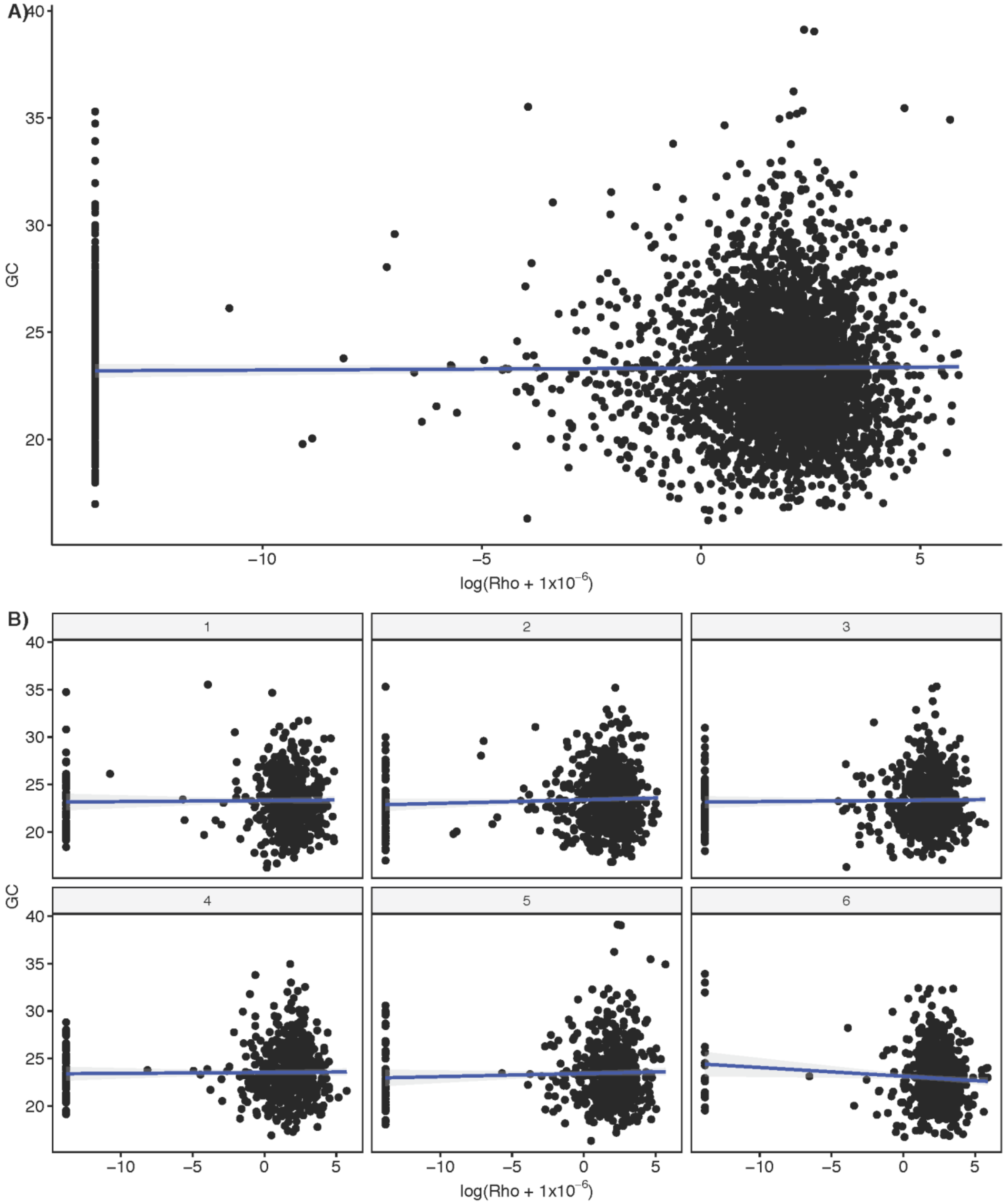

Figure S2 Relationship between the local recombination rate (Rho) inferred from natural strains and the local GC content (both on a log scale). **A)** The overall pattern across all locations in the genome and **B)** for the individual chromosomes. Plots include the intervals with zero inferred recombination events (which had a value of 10^{-6} added to allow for log transformation), but removal of these intervals does not change any of the relationships.

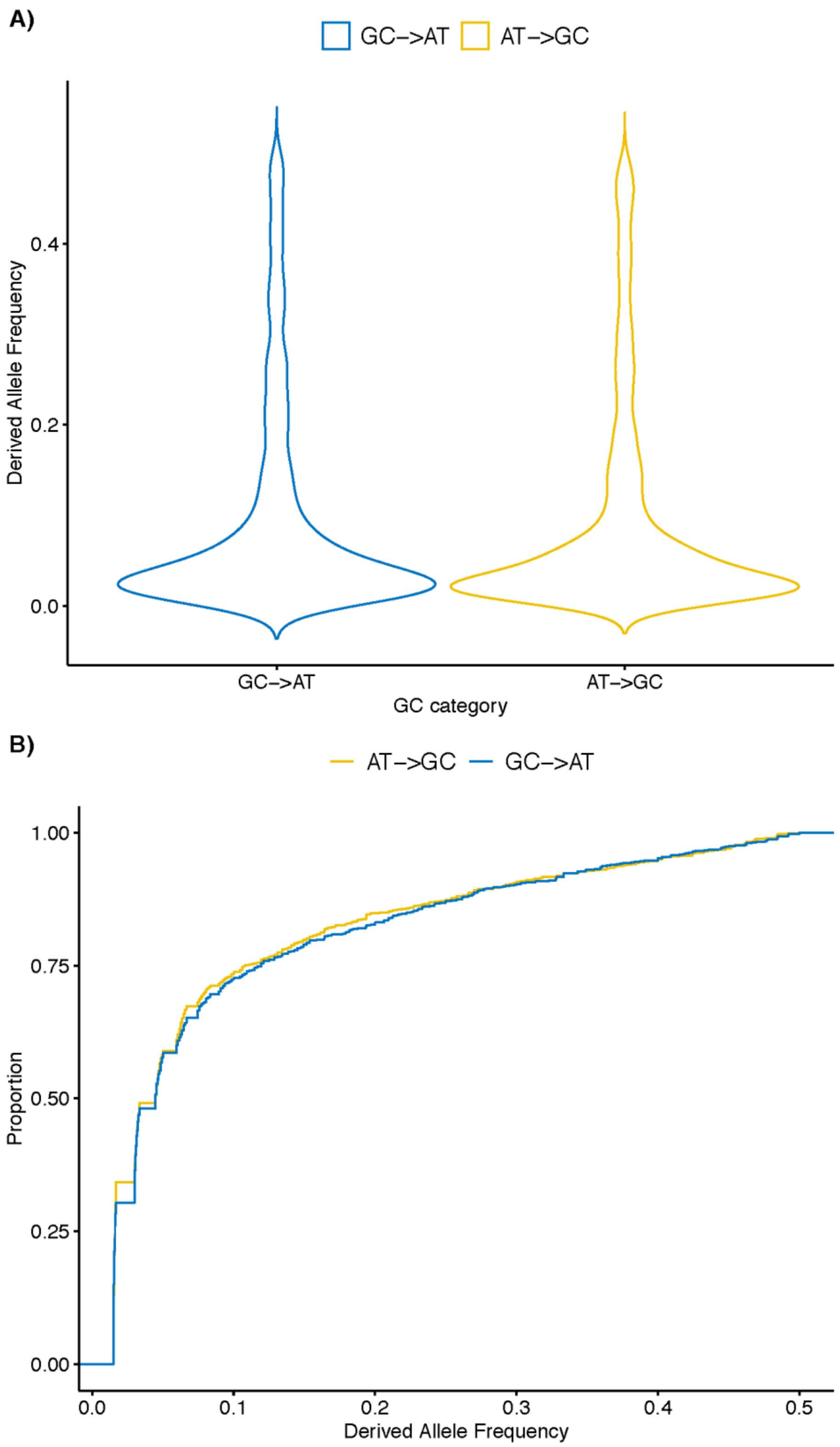

Figure S3. Frequency distribution of intronic SNPs corresponding to GC→AT or AT→GC changes. **A)** Violin plots of the frequency distribution of the derived allele for the two classes of SNPs. **B)** Cumulative proportion of loci across the derived allele frequency distribution (which corresponds to the distributions being compared in the Kolmogorov–Smirnov test).

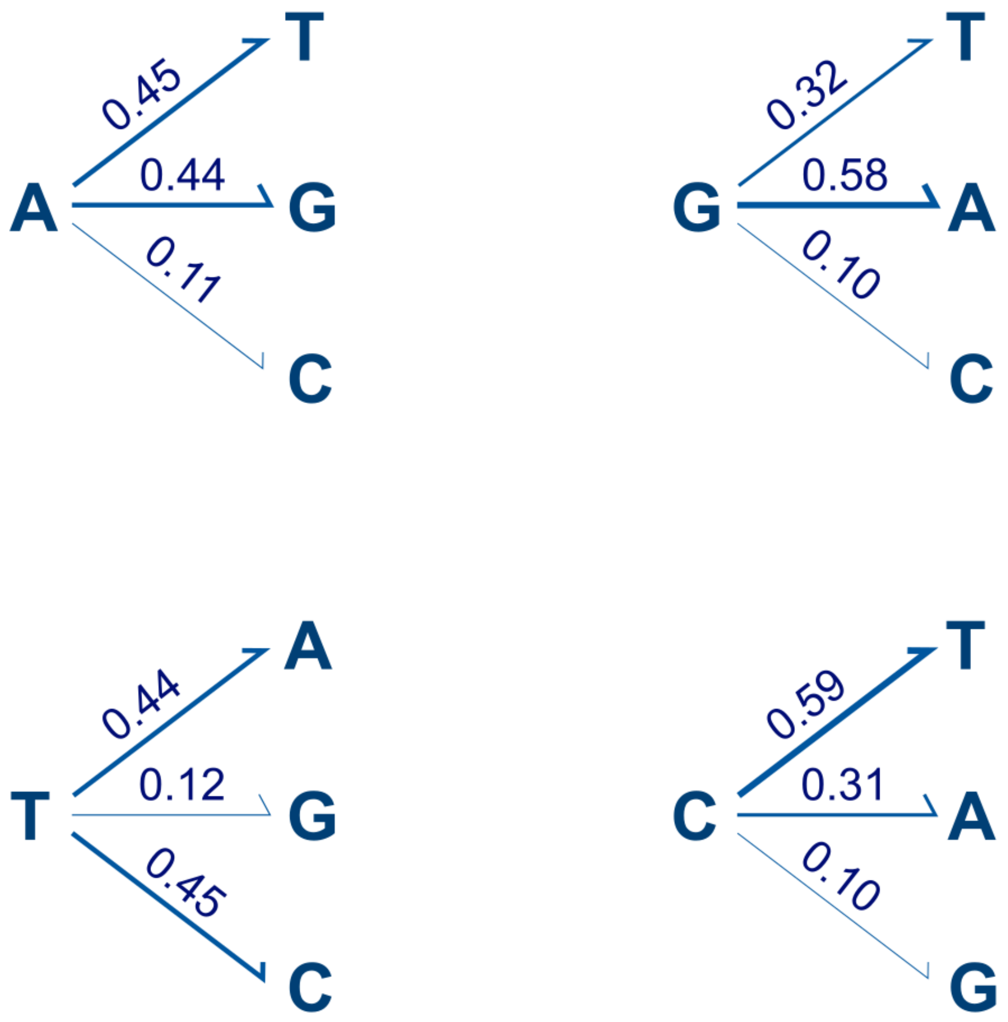

Figure S4: Relative nucleotide substitution matrix. Numbers indicate the fraction of substitutions (minor SNP class) towards each direction of the mutational space. Mutations are categorised as transitions, transversions and either following or against the overall bias towards AT accumulation (see text for more details).

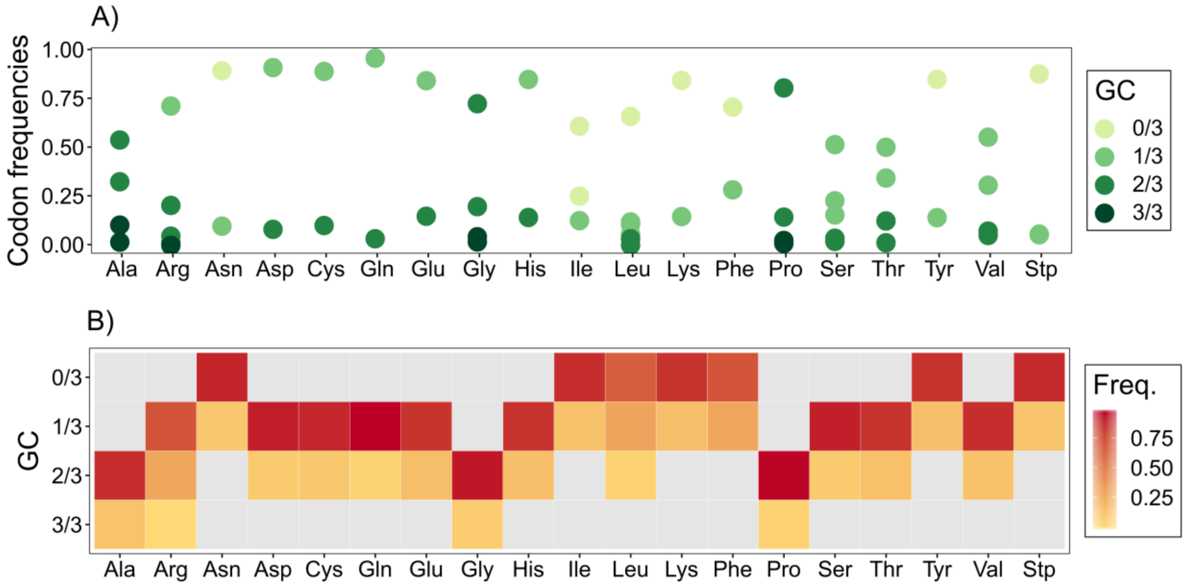

Figure S5. Relative codon frequencies and GC content. **A)** Proportional use of each codon for each amino acid and stop signal; points represent individual codons coloured according to their GC content. **B)** Pooled frequencies of synonymous codons with similar GC content per amino acid, with colours representing frequencies of codons (yellow: low; red: high) in each GC category.
